# Mycobacterium abscessus infection in the stomach of patients with various gastric symptoms

**DOI:** 10.1371/journal.pntd.0007799

**Published:** 2019-11-04

**Authors:** Deepak Chouhan, T. Barani Devi, Santanu Chattopadhyay, Sanjai Dharmaseelan, Gopinath Balakrish Nair, Krishnadas Devadas, Madhavan Radhakrishna Pillai

**Affiliations:** 1 Pathogen Biology Group, Rajiv Gandhi Centre for Biotechnology (RGCB), Thiruvananthapuram, India; 2 Manipal Academy of Higher Education (MAHE), Manipal, India; 3 Department of Gastroenterology, Government Medical College, Thiruvananthapuram, India; Beijing Institute of Microbiology and Epidemiology, CHINA

## Abstract

Development of gastric diseases such as gastritis, peptic ulcer and gastric cancer is often associated with several biotic and abiotic factors. *Helicobacter pylori* infection is such a well-known biotic factor. However, not all *H*. *pylori*-infected individuals develop gastric diseases and not all individuals with gastric diseases are infected with *H*. *pylori*. Therefore, it is possible that other gastric bacteria may contribute to the formation and progression of gastric disease. The aim of this study was to isolate prevalent gastric bacteria under microaerobic condition and identify them by 16S rRNA gene sequence analysis. Analysis of gastric biopsies showed infection of *Mycobacterium abscessus* (phylum Actinobacteria) to be highly prevalent in the stomachs of subjects included. Our data show that of 129 (67 male and 62 female) patients with gastric symptoms, 96 (51 male and 45 female) showed the presence of *M*. *abscessus* in stomach tissues. Infection of *M*. *abscessus* in gastric epithelium was further confirmed by imaging with acid fast staining, immunohistochemistry and immunofluorescence. Our imaging data strongly suggested that *M*. *abscessus* is an intracellular colonizer residing inside the gastric epithelial cells rather than in macrophages. Additionally, phylogenetic analysis of the mycobacterial *hsp65* gene showed that the nearest match to the *M*. *abscessus* strains isolated from our study subjects is the *M*. *abscessus* strain ATCC 19977. Surprisingly, the subjects studied, the prevalence of *M*. *abscessus* infection in stomach is even higher than the prevalence of *H*. *pylori* infection. This, to the best of our knowledge, is the first study showing the colonization of *M*. *abscessus* in human gastric mucosa among patients with various gastric symptoms. This study could provide usher in a new opportunity to understand the role of less studied gastric bacteria in the development of gastric diseases.

## Author summary

The development of stomach diseases like gastritis, ulcer and cancer are associated with several factors, which include the colonized stomach bacteria, lifestyle, the environment and the host genes. Soon after its discovery, the stomach bacterium *Helicobacter pylori* were linked with stomach diseases like gastritis, stomach ulcer and cancer. However not all *H*. *pylori* infected individuals develop stomach disease, and not all patients with stomach disease have *H*. *pylori* infection. Initially, *H*. *pylori* were thought to be the only relevant bacterial coloniser of the human stomach, but several studies have shown that bacteria other than *H*. *pylori* can also colonise in human stomach. The role of these bacteria remained unclear, but it has been shown that the bacterial diversity in stomach differs depending on whether *H*. *pylori* is present or not. This work illustrates that *Mycobacterium abscessus*, a non-tuberculous bacteria is among the most frequently observed gastric bacteria in individuals with various stomach diseases and it is important to evaluate the significance of this infection.

## Introduction

Development of gastric disease is dependent on several factors including *Helicobacter pylori* infection, host genotype, life style and perhaps, the gastric microbiome [1, 2]. *H*. *pylori*, a key member of the stomach microbiome, has been shown to be associated with gastric diseases such as gastritis, peptic ulcer and gastric cancer [3]. However, not all *H*. *pylori* infected individuals develop gastric diseases and not all individuals with gastric diseases carry *H*. *pylori* infection [4–6]. More than 50% of the world’s population may be infected with *H*. *pylori* and 10–20% of such infected individuals suffer from the above mentioned gastric diseases [7–9].

*H*. *pylori* is not the only bacterium that can colonize human stomach. Culture independent metagenomic sequence analyses have shown that human stomach carry a unique microbiota [10, 11]. The dominant phyla that are present in human stomach are Proteobacteria, Firmicutes, Actinobacteria and Fusobacterium [12]. Interestingly, however, most of these bacteria cannot be cultured using traditional techniques. Using culture based methods, the total bacterial count in human stomach may range from 0 to 103 cfu/g [13].

Presence of a diverse group of bacteria in stomach is not surprising since the stomach is exposed to food materials carrying microbial population. Indeed, nearly 65% of the phylotypes that have been identified in stomach have also been identified in the oral cavity [14]. Moreover, several bacteria such as *Streptococcus*, *Neisseria* and *Lactobacillus* are known for their acid tolerance and therefore can survive in low gastric pH. Conversely, presence of several bacteria (e.g. *Veillonella*, *Lactobacillus* and *Clostridium*) in gastric juice are possibly transient and it is not clear if they have any significant impact on the gastric epithelium [12, 15]. Interestingly, infection with *H*. *pylori* seems to have an impact in the gastric microbiome. For the healthy volunteers, not infected with *H*. *pylori*, *Streptococcus* and *Staphylococcus* are the main colonizers in the stomach [15, 16].

Description of gastric microbiota in Indian context is scanty. A recent population-based study on a cohort from Mumbai, showed the presence of several microbial genera in the stomach of dyspeptic and non-dyspeptic individuals [17]. Data from a recent metagenomic study on Mumbai population suggest that *H*. *pylori i*s likely to have negative interaction with members of the gastric microbiome while other gastric microbes are capable of having positive interactions among themselves [11]

Microbiome is known to vary with geography, ethnicity and food habits. Since India is a large country with multiple ethnic groups and diverse food habits, we expect to see difference in the composition of the gastric microbiota in regions other than Mumbai. For Trivandrum (the capital of the state of Kerala in Southern most part of India), there have been no studies that attempt to investigate gastric bacteria other than *H*. *pylori*. The primary aim of the current study was to isolate and identify prevalent gastric bacteria from a group of subjects visiting a referral hospital and evaluate their role in possible development of gastric disease.

## Results

### Isolation and identification of gastric *Mycobacterium abscessus*

**Fig 1:**
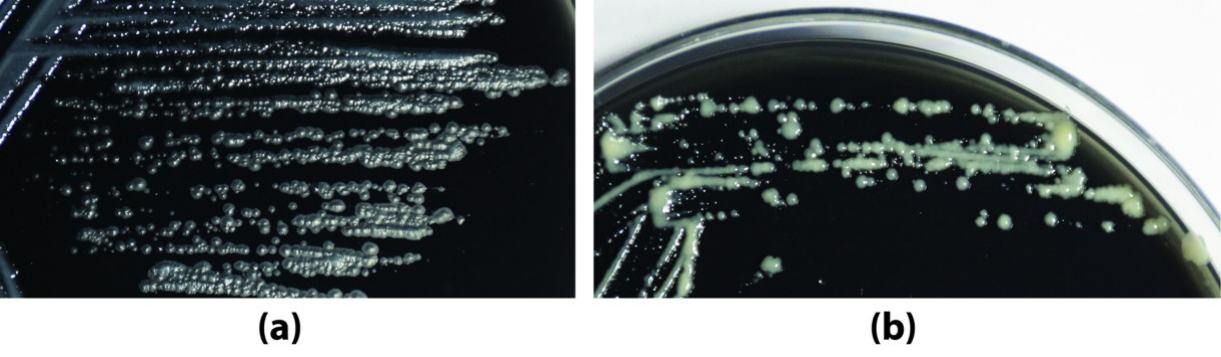
*M*. *abscessus* isolates. Isolated and sub-cultured rough (a) and smooth (b) *M*. *abscessus* colonies grown on BHI agar plates containing 7% calf serum and 0.4% charcoal after incubation at 37°C for 7 days in microaerobic condition.

Gastric biopsies collected in brucella broth were vortexed for 2 min, homogenized and 150 μl of the media containing the tissue lysates were inoculated on BHI blood agar plates (selective and non-selective). After incubating the selective and non-selective blood-agar plates in microaerobic condition for 5–7 days two types of bacterial colonies were observed and were further sub-cultured on BHI agar plates containing 7% calf serum and 0.4% charcoal. As shown in **Fig 1a and 1b**, a type of colonies appeared rough, big, white, and dry, while the other type of colonies appeared smooth, small, white, shiny and moist. For identification of these colonies, extracted DNA was used to amplify the bacterial 16S rRNA gene using primer sets designed to target the V1 to V5 variable regions. The PCR products were purified, sequenced directly using the BigDye terminator chemistry and subjected to BLAST analysis at NCBI platform. The small, white, shiny, smooth, moist colony was identified as smooth (S) variant and the bigger, white, rough, dry colony was identified as the rough (R) variant of *M*. *abscessus* (phylum: Actinobacteria). Both S and R variants of *M*. *abscessus* were preserved in BHI containing 20% glycerol at -80°C for further use.

### Confirmation that *M*. *abscessus* originate from gastric biopsies

**Fig 2:**
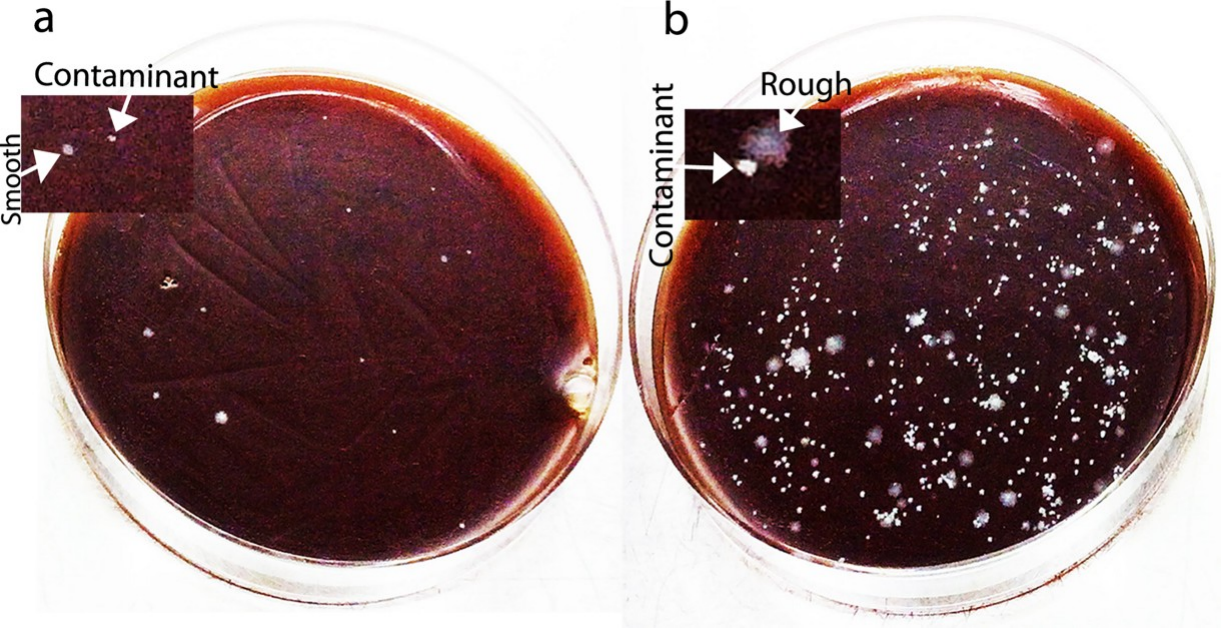
Confirmation of the gastric origin of *M*. *abscessus* by 3 wash assay. **(a)** The BHI blood agar plate that was inoculated with the biopsy without washing with PBS has fewer *M*. *abscessus* colonies. **(b)** The BHI blood agar plate that was inoculated with the biopsy that was washed in PBS 3 times and was homogenized before plating has numerous *M*. *abscessus* colonies.

*M*. *abscessus* is known to cause nosocomial infections. Therefore, it is not unlikely that the hospital borne *M*. *abscessus* strains may have contaminated the vials while collecting the gastric biopsies during endoscopy. To eliminate this possibility and to confirm that the collected gastric biopsy tissues are the real origin of the *M*. *abscessus* colonies that grew on blood agar plates, we washed the collected biopsies in 500 μl PBS for 3 times before homogenizing and inoculating on blood agar plates. Another biopsy specimen collected from the same patient was plated directly without the 3 wash and homogenization as control. As shown in Fig 2, when the biopsy was inoculated after 3 PBS washes and homogenization there are more number of *M*. *abscessus* colonies grown as compared to the biopsy that was not washed and not homogenized. This experiment confirms that the isolated *M*. *abscessus* strains are not a contaminant but originates from the biopsies. The experiment also suggests that the bacterium could possibly be intracellular since the number of colonies on the plate carrying the homogenized tissue is more than the plate carrying the non-homogenized tissue **(Fig 2)**.

### *erm*(41) gene PCR for *M*. *asbcessus* detection from gastric biopsy specimen

**Fig 3:**
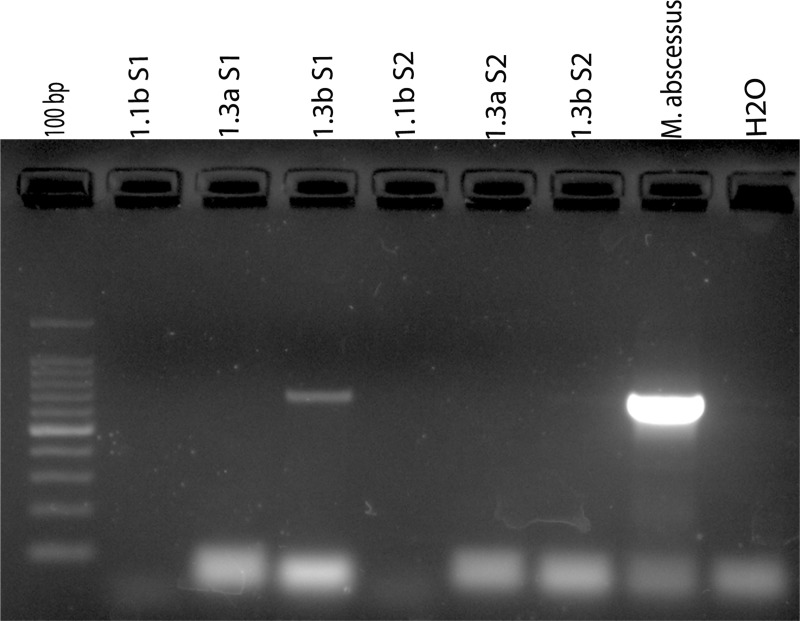
Detection of *M*. *abscessus* in gastric biopsy specimens using *erm*(41) gene PCR. Sample S1 (**1.3b S1**) shows amplification for *erm*(41) gene from the pellet but sample S2 (**1.3b S2**) shows no amplification from pellet. There is no amplification in PBS 1^st^ washed biopsy and supernatant collected after tissue homogenization (**1.1b S1 & S2 and 1.3a S1 & S2)**.

A modified method for isolating the intracellular bacterial DNA was used (details are given in Materials and Methods) to detect the *M*. *abcessus* colonized within the gastric epithelial cells. The biopsies collected in PBS were vortexed and centrifuged. The DNA extracted from the PBS did not give any amplicon for the PCR targeting the *M*. *abcessus erm*(41) gene. Then, the gastric biopsies were washed in PBS thrice followed by homogenization and centrifugation at 1300 rcf for 3 min. DNA was extracted from the supernatant and also from the pellet. When the DNA extracted from the supernatant was used no amplicon was obtained for the *erm*(41) gene. However, when the DNA extracted from the pellet was used, the presence of intracellular *M*. *abcessus* were detected as discerned by the same *erm*(41) gene PCR **(Fig 3)**.

### Prevalence of *M*. *abscessus* and *H*. *pylori* in the study population

In this study, a total of 65 *M*. *abscessus* strains were isolated from 129 patients **(Table 1)**. Of these 65 culture positive cases, we isolated both rough and smooth morphotypes of *M*. *abscessus* from 48 individuals, while 13 individuals were culture positive for only rough colonies and 4 individuals were culture positive for only smooth *M*. *abscessus* colonies. Of 129 patients, 96 were positive for *M*. *abscessus* for histological examination of gastric tissue sections using acid fast staining. Of 62 females recruited in the study 45 were *M*. *abscessus* positive and 17 were *M*. *abscessus* negative. Among 67 male, 51 were *M*. *abscessus* positive and 16 were *M*. *abscessus* negative (**Table 2)**.

**Table 1:** Prevalence of *M*. *abscessus* and *H*. *pylori* in the stomach of patients with various gastric diseases.

	Total	Age (20–30) Male-Female	Age (31–40) Male-Female	Age (41–50) Male-Female	Age (51–60) Male-Female	Age (61–70) Male-Female
**Total samples**	129	6–7	10–17	17–22	12–7	22–9
***M*. *abscessus* culture positive**	65	6–4	7–10	6–13	5–4	6–4
***M*. *abscessus* histology positive**	96	6–5	7–12	12–17	9–6	16–6
***H*. *pylori* culture positive**	17	1–0	3–2	3–2	1–1	2–2
***H*. *pylori ureB* PCR positive**	39	1–0	4–2	7–10	2–3	7–3

**Table 2:** Prevalence of gastric *M*. *abscessus* among male and female patients in Trivandrum.

Total samples	Male (67)		Female (62)	
**129**	***M*. *abscessus +ve***	***M*. *abscessus -ve***	***M*. *abscessus +ve***	***M*. *abscessus -ve***
	51	16	45	17

For the detection of *H*. *pylori* infection, only 17 of 129 patients were positive for culture when *H*. *pylori* specific media were used **(S2 Fig).** We also attempted to detect the presence of *H*. *pylori* in gastric biopsy specimens directly by PCR using primers specific for the *ureB* gene of *H*. *pylori* **(S1 Fig)** Since the PCR is more sensitive than culture, we were able to detect *H*. *pylori* infection for 39 patients by this method **(Table 1)**. Three patients were culture positive but *ureB* gene PCR negative. Overall, our study showed that of 129 patients, 96 (74.41%) were colonized by gastric *M*. *abscessus*, while only 42 (32.55%) were infected with *H*. *pylori*. (**Table 3).** Hence, for this study subjects from Trivandrum, the capital of the state Kerala in Southern India, the prevalence of *M*. *abscessus* infection is comparatively higher than the *H*. *pylori* infection in stomach.

**Table 3:** Prevalence of *M*. *abscessus* and *H*. *pylori* in the stomach of patients with various gastric diseases.

Total samples	*M*. *abscessus +ve* samples % (culture + histology)	*H*. *pylori +ve* samples% (culture + *ureB* PCR)
**129**	74.41% (96)	32.55% (42)

We also analyzed if patients carry both *M*. *abscessus* and *H*. *pylori* in their stomach. Among the 129 patients, 64 were infected with only *M*. *abscessus*, but not with *H*. *pylori*; 10 patents were infected with only *H*. *pylori*, but not with *M*. *abscessus*; and 32 patients were infected with both *M*. *abscessus* and *H*. *pylori* (**Table 4).** We then checked the prevalence of *M*. *abscessus* and *H*. *pylori* in patients with severe gastric diseases like peptic ulcer, gastric cancer and gastric polyp in their stomach. Gastric tissue pathology was done using H&E staining **(S3 Fig).** Of a total 25 patients who had severe gastric disease 13 were infected with *M*. *abscessus* but not with *H*. *pylori*; 11 were infected with *H*. *pylori* as well as *M*. *abscessus*; and one patient was infected with *H*. *pylori* but not with *M*. *abscessus* **(Table 5)**.

**Table 4:** Prevalence of co-infection of *M*. *abscessus* and *H*. *pylori* in Trivandrum.

*M*. *abscessus +ve* but *H*. *pylori* -*ve*	*H*. *pylori +ve* but *M*. *abscessus* -*ve*	*M*. *abscessus* and *H*. *pylori +ve* (total)
64	10	32

**Table 5:** Prevalence of *M*. *abscessus* and *H*. *pylori* among patients with severe gastric diseases.

Total samples	*H*. *pylori* and *M*. *abscessus +ve* samples	*H*. *pylori +ve* but *M*. *abscessus* -*ve* samples	*M*. *abscessus +ve* but *H*. *pylori* -*ve* samples
**25**	11	1	13

### *hsp65* gene PCR and phylogenetic analysis

**Fig 4:**
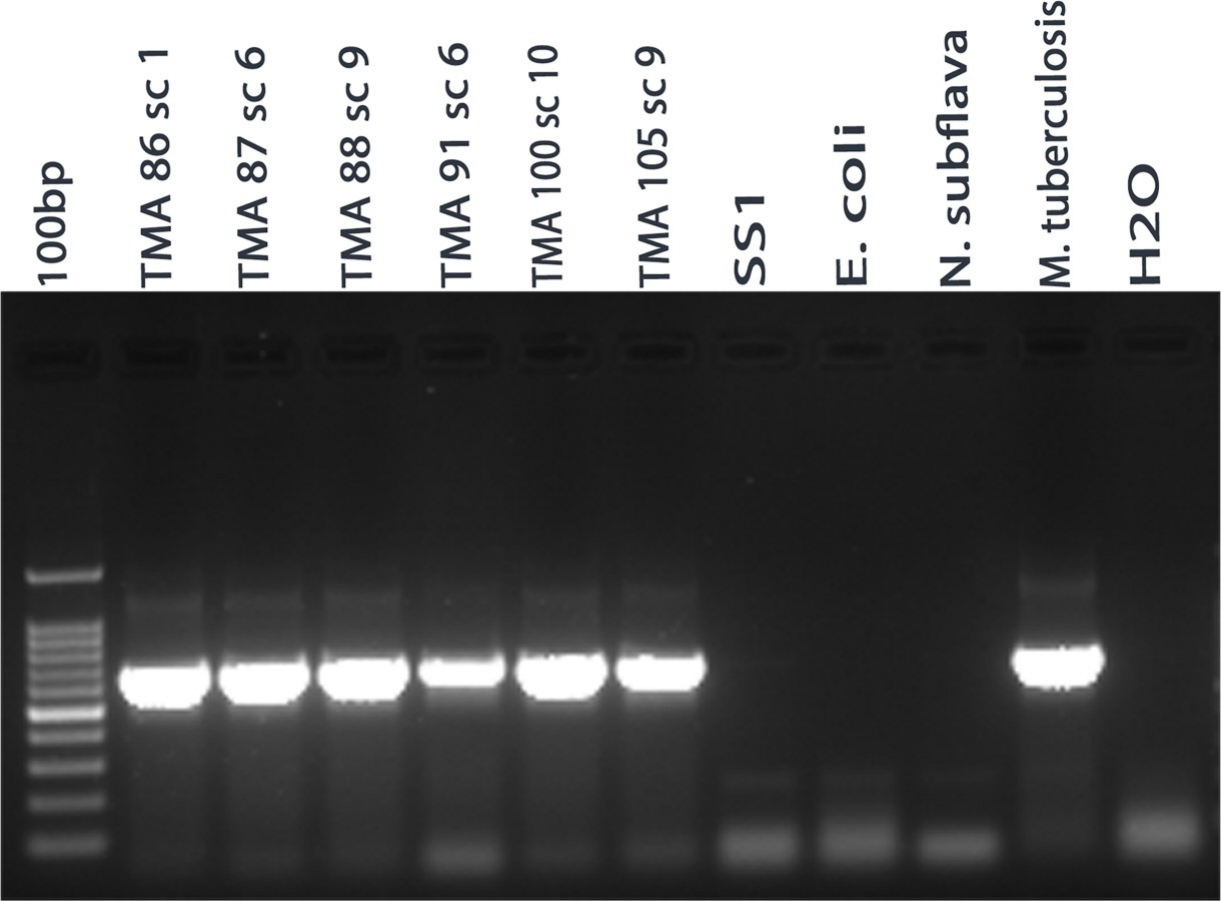
*hsp65* gene PCR analysis to confirm that the isolated strains belong to *Mycobacterium* genus. TMA 86 sc 1, TMA 87 sc 6, TMA 88 sc 9, TMA 91 sc 6 TMA 100 sc 10 and TMA 105 sc 9 showed the amplification for *hsp65* gene but *H*. *pylori*, *E*. *coli* DH5a and *N*. *subflava* gave no amplicon. DNA isolated from *M*. *tuberculosis* was used as positive control.

Colony morphology and 16S rRNA gene sequencing suggested that the isolated gastric bacterium is *M*. *abscessus*. For further confirmation, we wanted to verify if the isolated *M*. *abscessus* strains carry heat-shock protein 65 (*hsp65*) gene, since the *hsp65* gene is conserved thoughout the *Mycobacterium* genus. The DNA extracted from 36 randomly chosen *M*. *abscessus* strains were tested for the presence of *hsp65* gene by PCR. The *hsp65* gene was amplified by PCR for each isolated *M*. *abscessus* strains **(Fig 4)**. As expected, the *hsp65* gene was also amplified for the *M*. *tuberculosis* (positive control), while no amplicon was observed for *H*. *pylori* strain SS1, *E*. *coli* DH5α and *N*. *subflava* (negative controls).This experiment further confirmed that these gastric bacterial isolates (*M*. *abscessus*) belongs to the *Mycobacterium* group.

**Fig 5:**
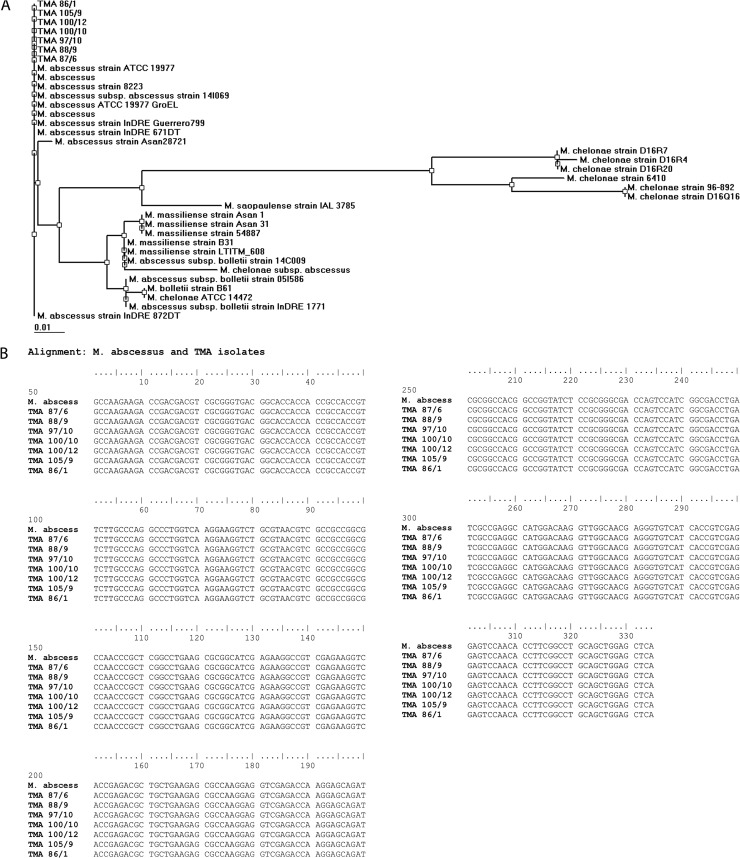
**(A**) **Phylogenetic tree based on *hsp65* gene sequence analysis of *M*. *abscessus* isolates.** TMA 86 sc 1, TMA 105 sc 9, TMA 100 sc 12, TMA 100 sc 10 TMA 97 sc 10, TMA 88 sc 9 and TMA 87 sc 6 shows these isolates are *M*. *abscessus* strain ATCC 19977. **(B) Sequence alignment of *M*. *abscessus* strain ATCC19977 and TMA isolates.** TMA 87 sc 6, TMA 88 sc 9, TMA 97 sc 10, TMA 100 sc 10, TMA 100 sc 12, TMA 105 sc 9, and TMA 86 sc 1 shows 100% sequence alignment with *M*. *abscessus* strain ATCC 19977.

A phylogenetic tree based on the *hsp65* gene sequence **(Fig 5A)** showed formation of monophyletic clusters of *M*. *abscessus*, *M*. *massiliense*, *M*. *chelonae* and *M*. *bolletii*. The *M*. *abscessus* strains isolated in this study from the human stomach was found to be closely related to the *M*. *abscessus* strain ATCC 19977 **(Fig 5A).** The nucleotide sequence of the *hsp65* genes of the gastric *M*. *abscessus* strains and the *M*. *abscessus* strain ATCC 19977 showed 100% identity **(Fig 5B)**.

### Acid-fast (Ziehl-Neelsen) and Immunohistochemistry staining of gastric tissue sections

**Fig 6:**
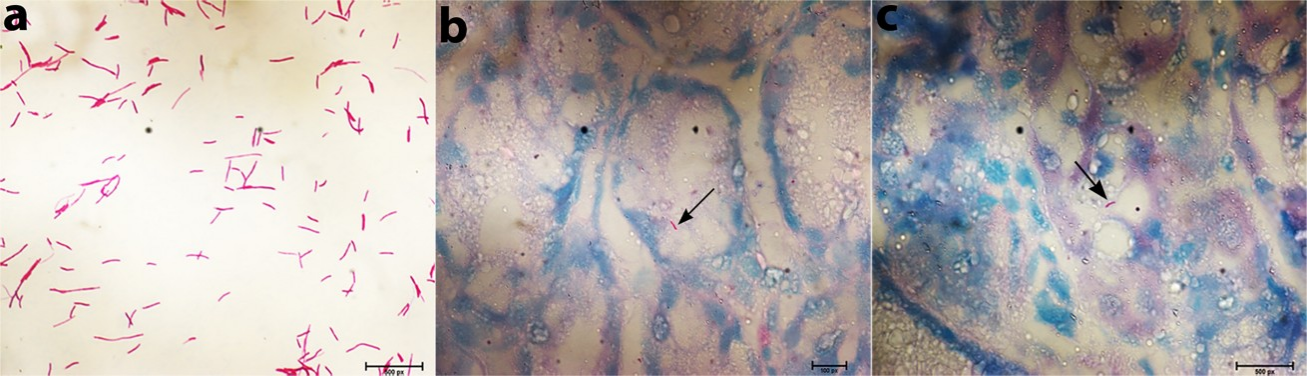
Identification of *M*. *abscessus* in gastric biopsy sections using Acid Fast (Ziehl-Neelsen) staining. **(a)** *M*. *abscessus* strains isolated from the gastric biopsies were subcultured, smeared on slides and were stained with Ziehl-Neelsen stain. **(b&c)** Ziehl-Neelsen staining of the two paraffin-fixed gastric tissue sections showed the presence of acid-fast bacilli (*M*. *abscessus*) at 100X magnification.

For the detection of *Mycobacterium*, the Acid-fast staining and Immunohistochemistry using the ab905 antibody against *Mycobacterium tuberculosis* PPD (purified protein derivative) are among the most reliable methods. To test if the isolated gastric *M*. *abscessus* strains are indeed acid fast bacilli (AFB), 23 representative strains were smeared on slides, heat killed and stained with Ziehl-Neelsen stain. All 23 strains were seen as acid fast bacilli under microscope at 100X magnification **(Fig 6a).** AFB were seen as beaded rods in association with mucosa and submucosa of gastric tissue, mostly in gastric gland bearing cells **(Fig 6b & 6c).** *M*. *abscessus* were identified in biopsies taken from antrum as well as from body of the stomach, suggesting that this bacterium is capable of colonizing all over the gastric mucosa. Of the 129 patients tested, AFB was detected in the gastric tissues for 96 patients by acid fast staining of paraffin fixed gastric tissue sections.

**Fig 7:**
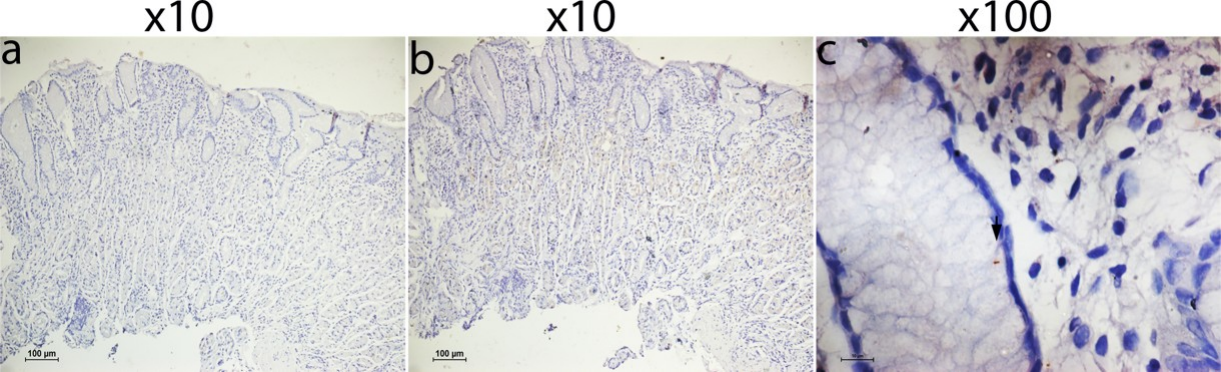
Identification of *M*. *abscessus* in gastric biopsy sections using Immunohistochemistry. **(a)** Immunohistochemistry of gastric biopsy section secondary antibody control without the primary antibody **(b)** Immunohistochemistry of gastric biopsy section with the ab905 antibody treated gastric biopsy section at 10X magnification. **(c)** Immunohistochemistry of gastric biopsy section using the ab905 antibody shows the presence of *M*. *abscessus* in the gastric tissue section at 100X magnification.

For the detection of this AFB in the gastric tissues by immunohistochemistry the ab905 antibody against *Mycobacterium tuberculosis* PPD was used. Tissue images were normalized with secondary antibody control without the primary antibody (-ab905) for background. The AFB were seen as beaded rods in association with mucosa and submucosa of gastric tissue, mostly in gastric gland bearing cells **(Fig 7c)**. No variation was noted in the number of bacilli (*M*. *abscessus*) in Acid-fast stained gastric tissue sections and antibody stained gastric tissue sections for the same gastric tissue sections.

### Immunofluorescence staining of gastric tissue sections

**Fig 8:**
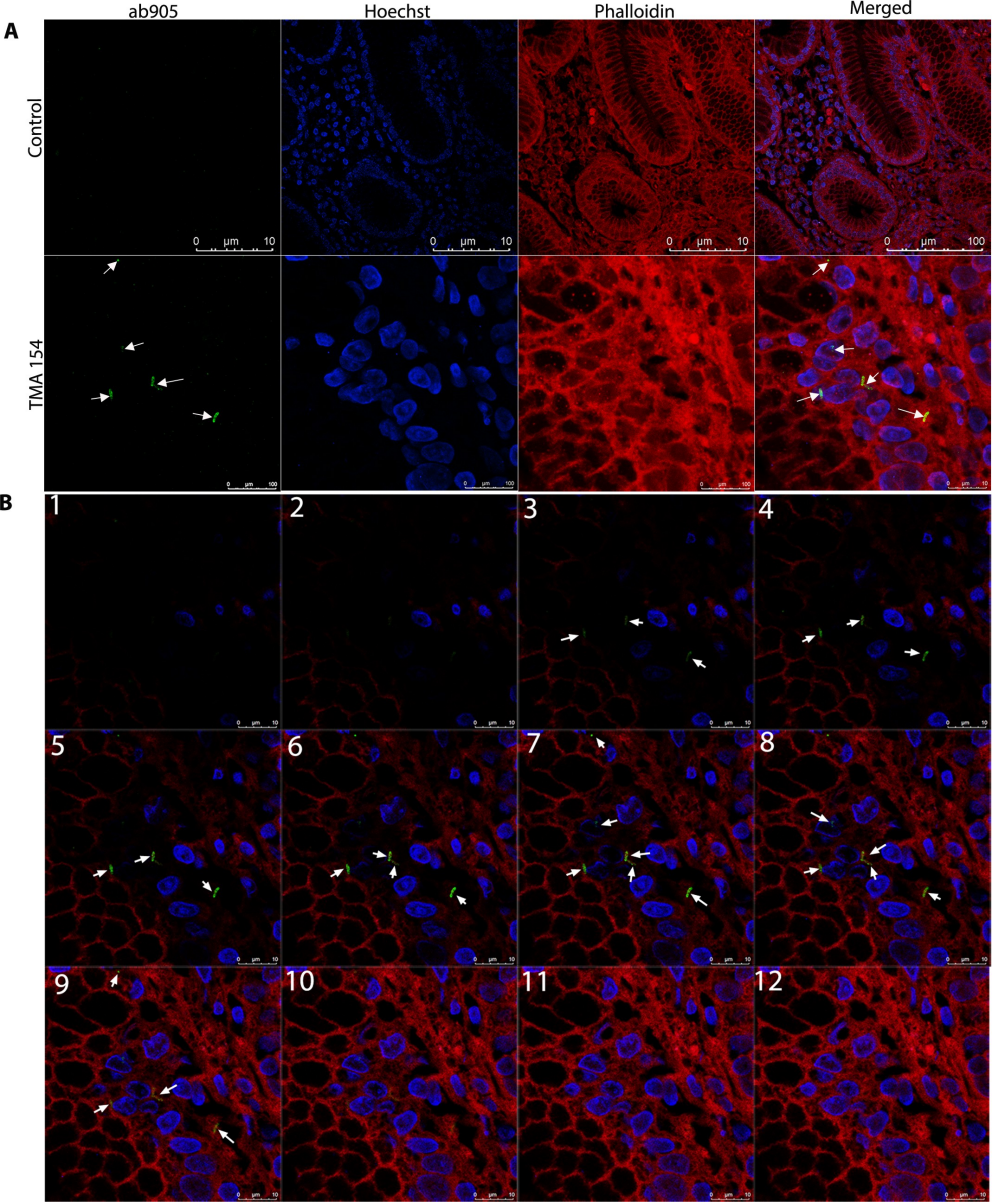
**(A) Immunofluorescence of gastric biopsy sections**. Paraffin-fixed tissue sections were subjected to confocal analysis using primary antibody + secondary antibody (ab905 + ab150077) for TMA 154 samples to stain *M*. *abscessus* and secondary alone (ab150077) as control. Confocal images show the presence of *M*. *abscessus* (green bacilli) in paraffin-fixed gastric tissue sections. **(B) Stacked images of gastric biopsy sections.** Stacked images (1–12) of paraffin- fixed gastric tissue sections show the presence of *M*. *abscessus* cells (green bacilli) within tissue section (3–9).

Both acid-fast and immunohistochemistry staining confirmed the presence of *M*. *abscessus* in gastric tissue sections. For further confirmation, direct immunofluorescence staining of gastric tissue section has been performed using primary antibody (ab905) and the alexa488 coated secondary antibody (ab150077). The confocal images were normalized with secondary antibody control without the primary antibody (-ab905) for background fluorescence. Confocal images show the presence of green rod shaped bacilli of *M*. *abscessus* in gastric tissues **(Fig 8A)**. The Z-stack and the 3D image of gastric tissue section further confirmed that this bacterium is intracellular **(Fig 8B).** *M*. *abscessus* has been seen mostly in the mucosa and submucosa of gastric tissue sections, particularly in gastric glands bearing cells as well as below the mucus layer attached to gastric epithelium. Like in immunohistochemistry, the *M*. *abscessus* cells were identified in gastric antrum as well as in gastric body by immunofluorescence.

## Discussion

*M*. *abscessus* is a non-tuberculosis mycobacteria (NTM), which has received recent attention due to its rapid growth and multidrug-resistance. It is known for being responsible for a wide spectrum of diseases related to skin and soft tissues, peritonitis, central nervous system infections and bacteremia in humans [18]. Interestingly, presence of this NTM in gastric juice was recently reported among cystic fibrosis patients [19]. Here, using various techniques like culture, 16S rRNA gene sequencing, *hsp65* gene sequencing, *erm*(41) gene PCR, acid-fast staining, immunohistochemistry and immunofluorescence we report that the infection of *M*. *abscessus* is fairly prevalent in our study subjects from a South Indian population with various gastric symptoms.

However, *M*. *abscessus* and other rapidly growing NTM have been well documented to cause nosocomial infections or hospital borne infections [20]. To eliminate this possibility all the medical devices that are used during endoscopy including the fiber optic endoscope and the biopsy forceps were well cleaned and treated with hypochlorite solution followed by 3 washing with water before every use. Therefore, the chances of obtaining the *M*. *abscessus* contamination through the medical devices are minimal. Furthermore, we have several lines of evidence showing that the origin of the isolated *M*. *abscessus* strains is indeed gastric tissues. First, when the gastric biopsies were washed 3 times and the tissues were homogenized before plating, the number of *M*. *abscessus* colonies grown on blood agar plates increased, indicating that the gastric biopsies are the true origin of the bacterium and is not a mere contaminant. Second, the confocal imaging data convincingly showed the presence of *M*. *abscessus* within the gastric epithelial cells and third, obtaining amplicon in *erm*(41) gene PCR from gastric biopsies. We also cultured the PBS on BHI plates as negative control but no bacterial colonies were observed on these plates **(S4 Fig).** All these evidences strongly support that the *M*. *abscessus* strains were isolated from the stomach of the patients suffering from gastric diseases and not hospital borne contaminants.

Although, *M*. *abscessus* is known to infect macrophages, our imaging data strongly suggest that the gastric *M*. *abscessus* is colonized mainly in the gastric epithelium, especially gastric mucosa and gastric gland bearing cells. A recent study has shown that *M*. *abscessus* can also infect and multiply in non-phagocytic cells like endothelial cells [21, 22]. Since the *M*. *abscessus*was seen under the mucus layer attached to gastric epithelium, it is possible that the bacterium gets colonized in the stomach through gastric lumen, possibly from food and water. However, once the bacterium penetrates the epithelium, it colonizes intracellularly (see Fig 2 and Fig 7). It is not clear at this point how *M*. *abscessus* passes through the gastric lumen, where the pH is highly acidic. However, the genome analysis of *M*. *abscessus* (NC_010397.1) shows that the bacterium carries urease gene, which may have a role similar to the urease gene of *H*. *pylori* in breaking down the gastric urea and creating a non-acidic microenvironment.

This, to the best of our knowledge, is the first report describing isolation and characterization of gastric *M*. *abscessus* among patients with gastric disorders. At present it is not understood if this bacterium has an impact on gastric diseases. However, *in vitro* studies have shown that *M*. *abscessus* activates mitogen-activated protein kinase and toll-like receptor-2 pathways and triggers secretion of pro-inflammatory cytokines and chemokines [23]. Also, in Zebrafish infection model, it has been shown that *M*. *abscessus* infection leads to the formation of aberrant granulomas, extensive mycobacterial cording, unrestricted bacterial growth and subsequent cell death as well as larval death [24, 25]. Few studies have shown that the rough variants of *M*. *abscessus* are more pathogenic and drug-resistant than the smooth variants [26, 27]. The rough variants of *M*. *abscessus*is hyper lethal for C57BL/6 mice as it induces a strong tumour necrosis factor alpha response by murine monocyte-derived macrophages [28]. Interestingly, we have isolated both rough and smooth variants of the *M*. *abscessus* from the gastric tissue specimens.

It is also interesting to note that prevalence of gastric *M*. *abscessus* in our Trivandrum study subjects is significantly high, even higher than the prevalence of *H*. *pylori* infection. We found that appxomately 74.41% of individuals with gastric diseases were infected with *M*. *abscessus*, where 96 out of 129 (67 male and 62 female) biopsy specimens carry *M*. *abscessus* infection in their stomach, while only 42 samples (32.55%) were found to be infected with *H*. *pylori*, indicating that the prevalence of gastric *M*. *abscessus* is higher than the prevalence of *H*. *pylori* infection in these subjects. Of 96 *M*. *abscessus* infected samples 51 were male and 45 were female, suggesting no significant difference in the prevalence of *M*. *abscessus* infection between male and female. We also isolated *H*. *pylori* and *M*. *abscessus* from 25 patients (6 female and 19 male) with severe gastric disease. Of them, 13 were infected with *M*. *abscessus* but not with *H*. *pylori*, while 11 patients were infected with both *H*. *pylori* as well as *M*. *abscessus*. One patient was infected with only *H*. *pylori* and not with *M*. *abscessus*. This suggests that approximately 96.00% of individuals with severe gastric disease were infected with *M*. *abscessus*.

In conclusion, our study on 129 individuals with gastric diseases shows that the prevalence of gastric *M*. *abscessus* is higher in the local population as compared to the prevalence of *H*. *pylori*. The route of transmission is not known at present, but water could be a source. Significance of this infection is also presently unknown, but it may have a significant role in the formation or progression of gastric disease. Further studies involving *in vitro* and *in vivo* host-microbe interaction is needed to understand the significance of *M*. *abscessus* in gastric mucosa.

## Methodology

### Ethics statement

The study was approved by the Human Ethics Committee of the Trivandrum Medical College (Approval Number IEC.No.05/07/2016/MCT) and Institute Human Ethics Committee of Rajiv Gandhi Centre for Biotechnology (Approval Number IHEC/01/2017/18). All the patients (aged between 20 and 70) were given written informed consent before enrollment and signed consent was obtained from all participants. There were no child participants in this study.

### Study population and sample collection

Samples were collected at the Medical College, Trivandrum, Kerala, India. Trivandrum is the most Southern part of India, mostly the part of western ghats with high humidity. Patients recruited for this study were mostly non-vegetarian, their food included fermented rice based food, fish, meat, egg and vegetables. Trivandrum sub urban region mostly drinks water from wells. Patients aged between 20 and 70 with various gastric and esophageal symptoms ranging from mild dyspepsia, gastro esophageal reflux disorder to severe gastric diseases like gastric cancer and who were recommended to have upper gastrointestinal endoscopy, were recruited in the study. Patients who had active gastric bleeding, coagulopathy as well as those who were on treatment with antibiotics or proton pump inhibitors within two weeks prior to endoscopy, were excluded from the study. Three gastric biopsy specimens were collected from each patient for this study. One biopsy was collected in Brucella broth containing 20% glycerol, another collected in phosphate buffered saline (PBS) and the third one was collected in 4% formaldehyde solution.

### Gastric bacteria culture

Brain Heart Infusion (BHI) agar plates containing defibrinated sheep blood (7%) and 0.4% IsoVitaleX (Becton&Dickinson, Sparks, MD, USA) were used for isolation of the gastric bacteria. While specifically isolating *H*. *pylori*, the Dent (Oxoid LTD, Basingstoke, Hampshire, England) selective supplement was added to make the media selective for *H*. *pylori*. The biopsy samples were homogenized and inoculated on both selective and non-selective blood agar plates and were incubated at 37°C in microaerobic condition (5% O_2_, 10% CO_2_, 85% N_2_).

### Three wash assay

**Fig 9:**
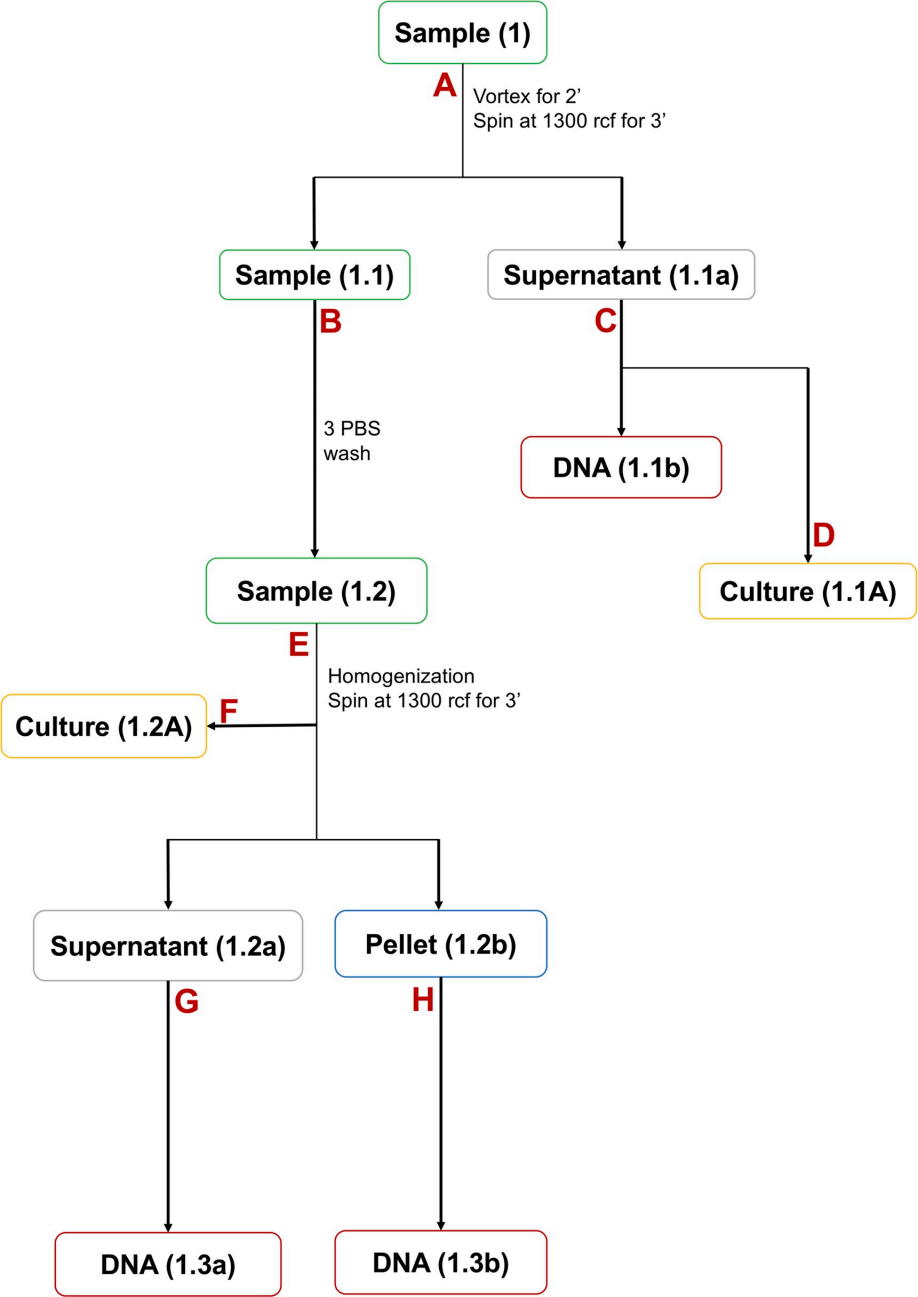
Work flow description of bacterial culture and DNA isolation from biopsies. **(A)** In this step biopsies taken in PBS were vortexed and centrifuged at 1300 rcf for 3 min to remove environmental contaminant from the biopsy. (**B)** Biopsies were washed thrice with PBS to remove all possible environmental contaminant. **(C)** DNA were isolated from supernatant (1^st^ washed PBS). **(D)** Supernatant (1^st^ washed PBS) were cultured on BHI plates for environmental contaminants. **(E)** 3 washed biopsies were homogenized (single cell suspension) and centrifuged at 1300 rcf for 3 min to remove extracellular bacteria (supernatant) from the gastric epithelial cells to get intracellular bacteria (pellet). **(F)** 3 washed biopsies were homogenized and cultured on BHI plates for intracellular bacteria. **(G & H)** Bacterial DNA were isolated from the supernatant (extracellular bacteria) and from the pellet (intracellular bacteria).

The biopsy samples were taken in 200 μl PBS and centrifuged at 1300 rcf for 3 min. The PBS was discarded by pipetting and 500 μl fresh PBS was added. After inverting, the biopsy containing tubes were centrifuged again at 1300 rcf for 3 min and the supernatant removed by pipetting. The same procedure was repeated twice more (3 times in total) and 500 μl of PBS was added in each tube containing the washed biopsy. The tubes were vortexed for 2 min and the biopsies were then homogenized. From this homogenized suspension, 150 μl was inoculated on BHI blood agar plates and the plates were incubated in microaerobic condition at 37°C for 7–13 days (**Fig 9**).

### Bacterial DNA isolation

The bacterial DNA was isolated as previously described [29]. In brief, the bacterial colonies were harvested in 500 μl PBS and centrifuged at 5,000 rcf for 10 minutes. The bacterial pellet was resuspended in 200 μl GTE buffer. The bacterial suspension was then treated with lysozyme (10 mg/ml) at 37°C for 1 hour. After enzymatic digestion, bacterial cells were lysed using TES buffer. Proteinase K (50 μg/ml) and RNase (20 μg/ml) were then addedand tubes were incubated at 55°C for 2 hours. The digested bacterial proteins were removed by phenol: chloroform: isoamyl alcohol and then by chloroform: isoamyl alcohol treatments. The bacterial DNA was precipitated using 3M sodium acetate (pH 5.2) and chilled ethanol.

### Extraction of bacterial DNA from biopsy tissue

Bacterial DNA from the stomach biopsy was isolated as described elsewhere [30, 31]. In brief, the gastric biopsy specimen was taken in PBS and vortexed for 10 minutes. The bacteria were lysed by a combination of enzymatic, chemical as well as mechanical methods. Finally, the DNA was precipitated using chilled absolute ethanol.

### Extraction of intracellular bacterial DNA from biopsy tissues

To make single cell suspension of the gastric cells, the gastric biopsies were homogenized (using a tissue homogenizer) and were centrifuged at 1300 rcf for 3 min. The supernatant and the pelleted cells were taken in different tubes and were incubated with proteinase K (100μg/ml) solution at 50°C for overnight. After overnight incubation two sterilized glass beads (2.5mm) were added and the tubes were vortexed for 10 min for mechanical rupture of the gastric cells so that the intracellular bacteria get released. For the isolation of bacterial DNA, the protocol that was described above was followed (**Fig 9**).

### Molecular characterization of the bacteria isolated from stomach

For the detection of *H*. *pylori* in gastric biopsies, DNA extracted directly from gastric biopsies were used for PCR to amplify the *ureB* gene of *H*. *pylori* [32] and for the detection of *M*. *abscessus*, *erm*(41) gene was targeted [33]. For the identification of isolated gastric bacteria (other than *H*. *pylori*), a 916 bp amplicon covering the V1 to V5 regions of the bacterial 16S rRNA gene was generated by PCR using specific primers **(Table 6)**. The PCR products were purified using QIAquick PCR purification kit (Qiagen, Hilden, Germany) and were used for sequencing reaction using the BigDye termination v3.1 cycle sequencing kit (Thermo Fisher Scientific, Waltham, Massachusetts, US). The products of the sequencing PCR were purified by ethanol precipitation and 70% ethanol wash. Finally, the purified DNA fragments were sequenced by a 3730XL DNA analyser (Thermo Fisher Scientific, Waltham, Massachusetts, US). The identities of the bacterial species were determined by BLAST.

**Table 6:** 

Gene Name	Primer Name	Sequence	Reference
*ureB*	ureB-F ureB-R	F 5’-CGT CCG GCA ATA GCT GCC ATA GT-3 R 5’-GTA GGT CCT GCT ACT GAA GCC TTA-3	[32]
16S rRNA gene	V3-V5F2 V3-V5R2	F 5’-GCC TAC GGG AGG CAG CAG-3’ R 5’-ATT ACC GCG GCT GCT GG-3’	This study
*hsp65*	HSPF3 HSPR4	F 5'-ATCGCCAAGGAGATCGAGCT-3' R 5'-AAGGTGCCGCGGATCTTGTT-3'	[34]
*erm*(41)	ermF ermR1	F 5'-GACCGGGGCCTTCTTCGTGAT-3' R 5'-GACTTCCCCGCACCGATTCC-3'	[33]

The identified *Mycobacterium abscessus* strains were further characterized by amplifying the *hsp65* gene by PCR and sequencing followed by phylogenetic analysis [34]. In brief, the sequences were assembled using BioEdit software (version 7.2.6.1) and multiple sequence alignments were carried out using ClustalW. A phylogenetic tree based on the *hsp65* gene sequences was constructed using the BioEdit program.

### Hematoxylin and Eosin (H&E) staining

The gastric tissues embedded in paraffin blocks were sectioned (5 μm) using a Leica Leitz 1512 microtome (Leica Microsystems, Nussloch, Germany). Deparaffination of the tissue sections were done using xylene (3 × 10 min), followed by rehydration with isopropanol in decending order (100%, 95%, 70%, 50%) and finally with water. Then, the tissue sections were stained with Hematoxylin (Himedia) for 30 min followed by acid alcohol wash and bluing with mild alkaline water (0.2% ammonium hydroxide). The sections were then dipped in 2% Eosin (Himedia) for 40 sec followed by washing and mounting. The stained sections were examined under a Nikon Eclipse 55i microscope (x10 and x20).

### Acid-fast staining (Ziehl-Neelsen stain)

Acid-fast staining was performed according to slandard protocol [35, 36]. The paraffin embedded gastric tissue sections (5 μm) were deparaffinized and rehydrated as mentioned above. After rehydration, the tissue sections were stained by the addition of carbol-fuchsin (Himedia) and heating till fumes started to appear. Then the sections were allowed to cool down for 10 min and were washed with distilled water. The sections were then decolorized by acid alcohol and washed with distilled water. Finally, the sections were counterstained with methylene blue for 30 sec, air dried and mounted for examination under microscope.

### Immunohistochemistry

The 5 μm gastric tissue sections were deparaffinized as mentioned above and was partially rehydrated using 100%, 95% and 80% isopropanol. The endogenous peroxidase activity was inhibited by 0.03% hydrogen peroxide (H_2_O_2_) in 70% methanol for 10 minutes. The sections were then treated with 50% isopropanol followed by water to completely rehydrate the tissue sections. Antigen retrieval of gastric tissue sections were done using sodium citrate buffer (pH 6.0) at 95°C for 20 min, followed by blocking of nonspecific antigens with 3% Bovine Serum Albumin (BSA) in Tris-buffered saline (TBS). The sections were then incubated with a rabbit polyclonal antibody, ab905 (Abcam, Cambridge, UK) against *Mycobacterium tuberculosis* PPD (purified protein derivative) at 1:500 dilutions in TBS containing 1% BSA for overnight in a humified chamber at 4°C. After washing with Tris-buffered saline containing 0.1% tween 20 (TBST), the sections were incubated for 1 hour with secondary antibody conjugated to horseradish peroxidase (HRP), followed by addition of substrate system (Dako REAL EnVision Detection system, Peroxidase/DAB+, Rabbit/Mouse K5007, Denmark). Sections were washed with TBST and stained with hematoxylin (Sigma-Aldrich) for visualizing nuclei and mounted in DPX (Di-N-Butyl Phthalate in xylene, Merck). The immune stained images were quantified by using Nikon NIS elements software (Japan). The relative intensities of staining in gastric tissue sections were corrected for background and normalized using the secondary antibody control without the primary antibody (-ab905).

### Immunofluorescence

For immunofluorescence, the gastric tissues were sectioned (15 μm), deparaffinized, rehydrated and the endogenous peroxidase activity was inhibited as mentioned above. Antigen retrieval was also done as mentioned above (Immunohistochemistry section). The gastric tissue sections were permeabilized using PBS containing 0.25% Triton X-100 (Sigma-Aldrich, Missouri, US) for 10 min and the tissue sections were washed with PBST (PBS + 0.1% Tween 20). Blocking of nonspecific antigens was done with 3% BSA in PBST for 10 min. The primary antibody ab905 was used in 1:300 dilutions with 1% BSA in PBST. After overnight incubation with the primary antibody in humidified chamber at 4°C, the tissue sections were washed with PBST. The tissue sections were incubated with Alexa flour 488 coated secondary antibody ab150077 (Abcam, Cambridge, UK) at 1:1000 dilutions in PBST containing 1% BSA for 1 hour. The sections were then washed with PBST, followed by incubation for 3 min with Phalloidin (Sigma-Aldrich, Missouri, US). The gastric tissue sections were then washed with PBST for 3 times. Finally, the tissue sections were incubated with 10 μg/ml Hoechst (Sigma-Aldrich, Missouri, US) for 1 min to stain the nuclei of the cells. Finally, the sections were mounted for confocal microscopy.

## Supporting information

S1 Fig Detection of *H*. *pylori* in gastric biopsy specimens. *H*. *pylori* specific *ureB* gene PCR amplicons confirm that TMA 120 is *H*. *pylori* positive and rest other TMA 119,121 and 122 are negative for *H*. *pylori*. 26695 (*H*. *pylori* reference strain) DNA has been used as a positive control and water and *E*. *coli* DH5a as negative control.
(TIF)

S2 Fig Gastric bacterial culture from gastric biopsy specimen. Gastric biopsy specimens were cultured on BHI blood agar plates for the isolation of gastric bacteria. *H*. *pylori* reference strain SS1 were cultured on BHI plates, the appearance of *H*. *pylori* colonies are transparent, shiny like a water droplets. Gastric bacteria other than *H*. *pylori* can be identified by the colony appearance, morphology, pigmentation etc. Gastric bacteria *H*. *pylori* and others are grown on the BHI plates, which were cultured using gastric biopsy specimens.
(TIF)

S3 Fig Hematoxylin and Eosin staining. Paraffin fixed gastric tissue sections were stained with Hematoxylin and Eosin and images were taken using Nikon microscope. 10X and 20X magnification. Gastric tissues sections show that accumulation of lymphocytes as well as disruption of gastric mucus layer.
(TIF)

S4 Fig Bacterial culture from PBS. 0.22μm membrane passed and autoclaved PBS was cultured on BHI serum + charcoal and serum plate, but no bacterial colonies were observed.
(TIF)
