# Supplementary figures and images for: Mycobacterium abscessus infection in the stomach of patients with various gastric symptoms
Source: PLoS Negl Trop Dis. 2019 Nov 4;13(11):e0007799. doi: 10.1371/journal.pntd.0007799 (PMC6855505; doi:10.1371/journal.pntd.0007799)

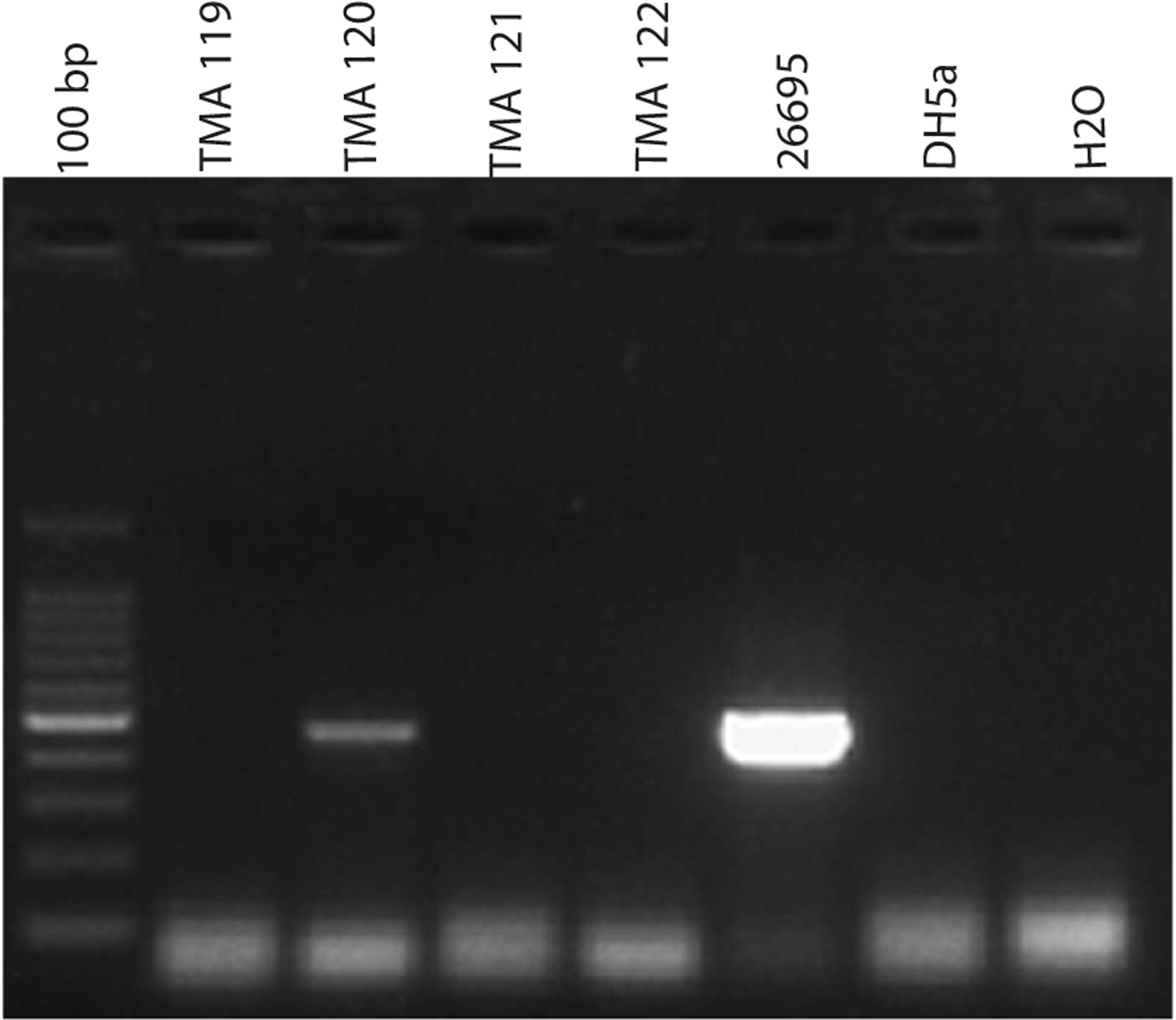

Supplement: S1 Fig — H. pylori specific ureB gene PCR amplicons confirm that TMA 120 is H. pylori positive and rest other TMA 119,121 and 122 are negative for H. pylori. 26695 (H. pylori reference strain) DNA has been used as a positive control and water and E. coli DH5a as negative control. (TIF) [file pntd.0007799.s001.tif]

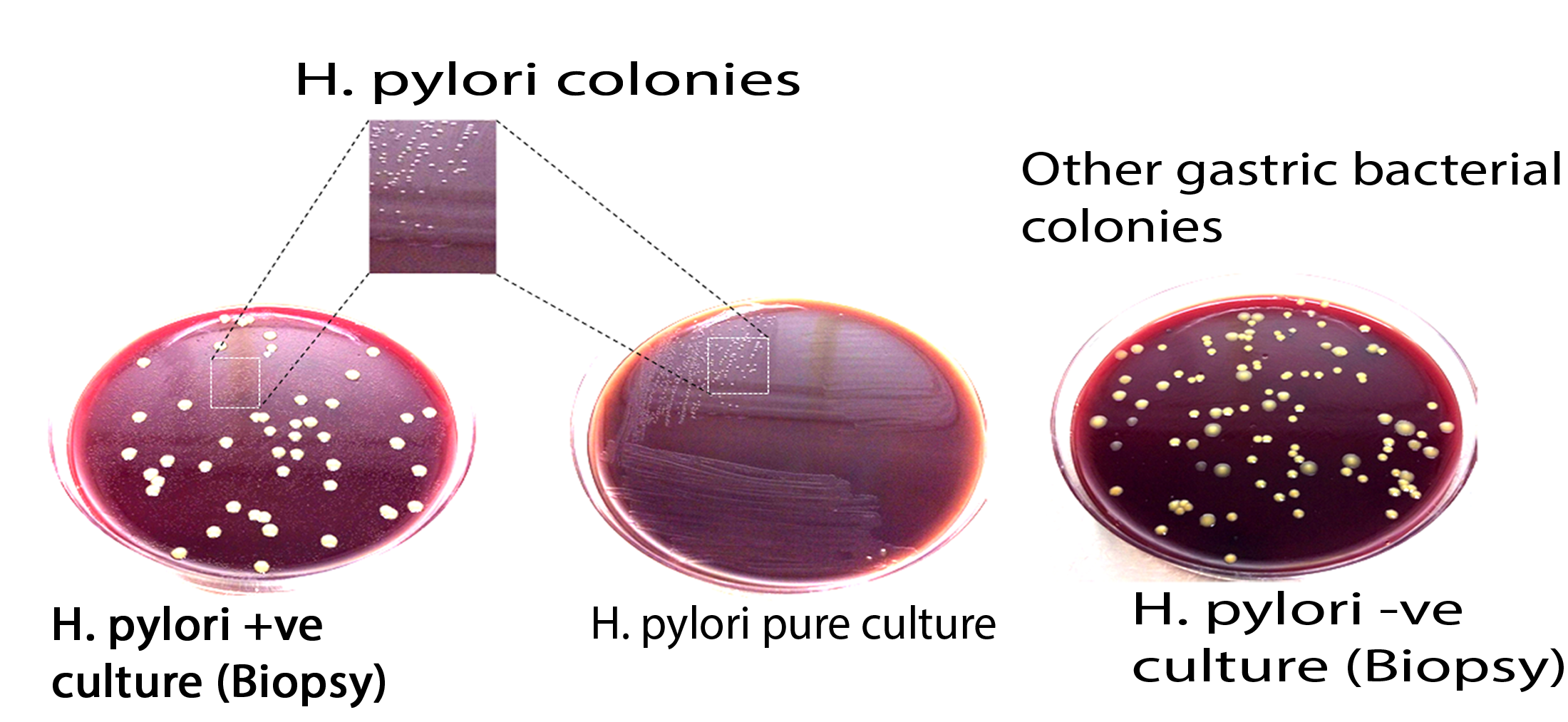

Supplement: S2 Fig — Gastric biopsy specimens were cultured on BHI blood agar plates for the isolation of gastric bacteria. H. pylori reference strain SS1 were cultured on BHI plates, the appearance of H. pylori colonies are transparent, shiny like a water droplets. Gastric bacteria other than H. pylori can be identified by the colony appearance, morphology, pigmentation etc. Gastric bacteria H. pylori and others are grown on the BHI plates, which were cultured using gastric biopsy specimens. (TIF) [file pntd.0007799.s002.tif]

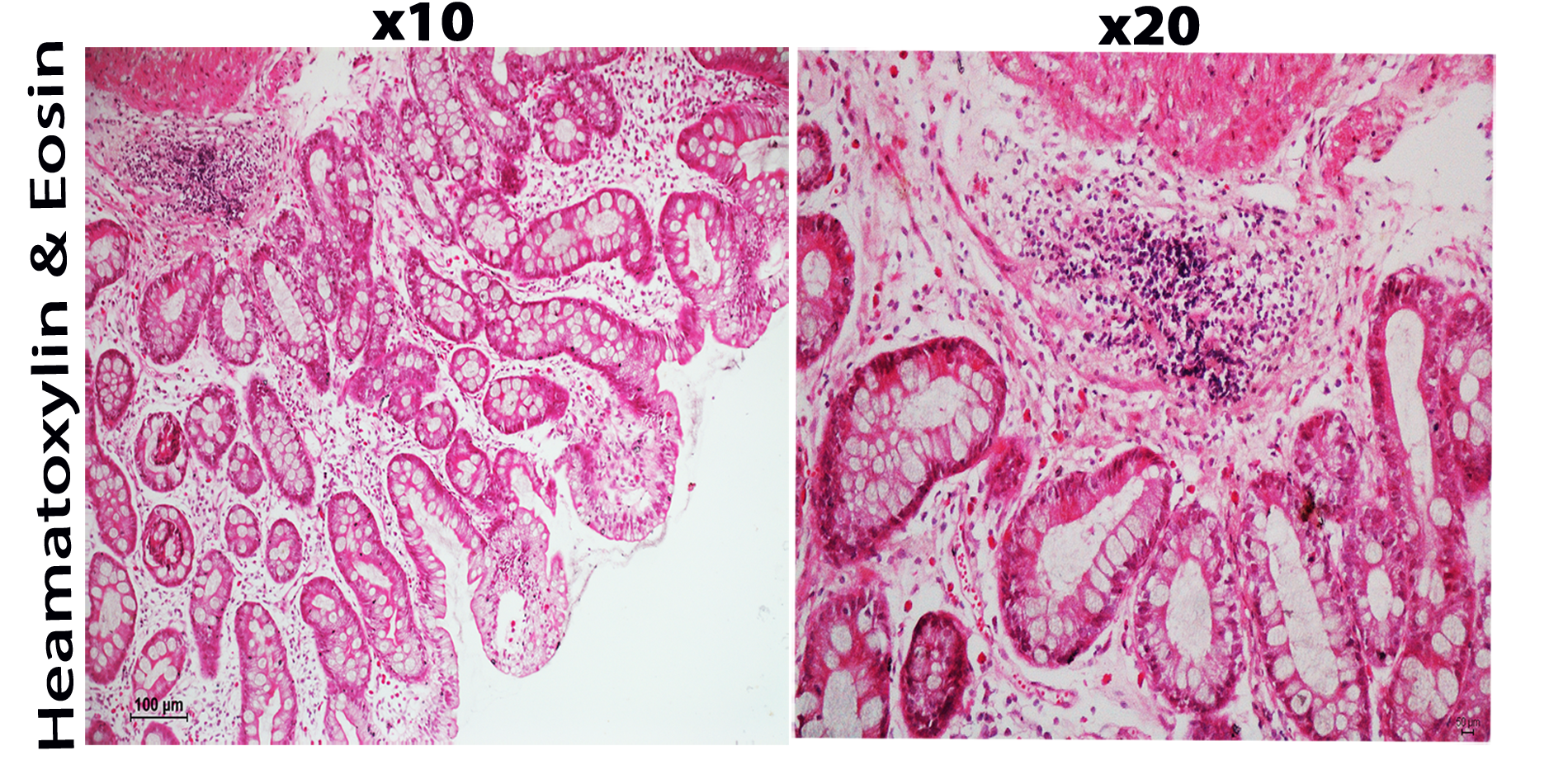

Supplement: S3 Fig — Paraffin fixed gastric tissue sections were stained with Hematoxylin and Eosin and images were taken using Nikon microscope. 10X and 20X magnification. Gastric tissues sections show that accumulation of lymphocytes as well as disruption of gastric mucus layer. (TIF) [file pntd.0007799.s003.tif]

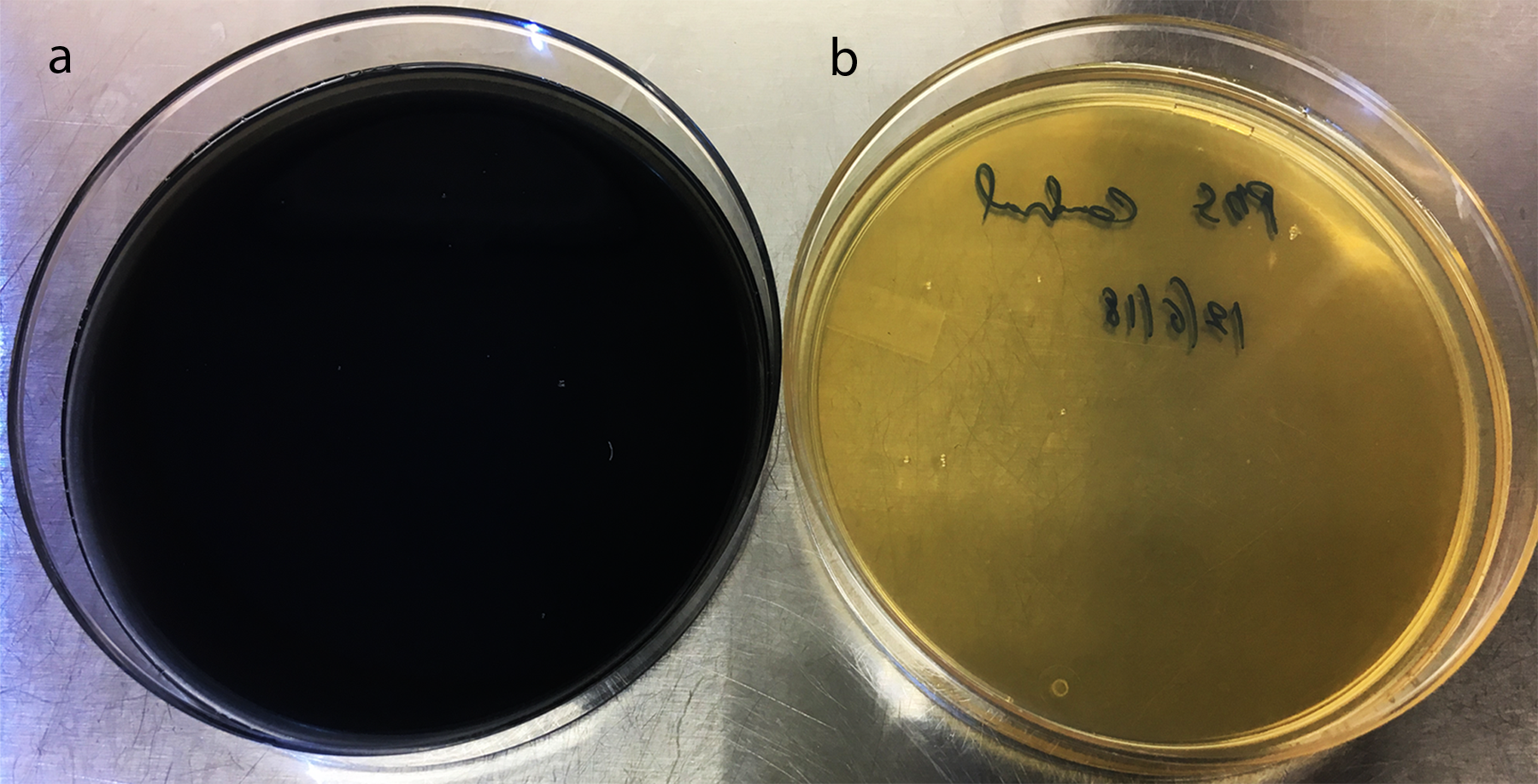

Supplement: S4 Fig — 0.22μm membrane passed and autoclaved PBS was cultured on BHI serum + charcoal and serum plate, but no bacterial colonies were observed. (TIF) [file pntd.0007799.s004.tif]
